# Passive Motion

**Published:** 1894-10-06

**Authors:** 


### PASSIVE MOTION.

The principal function of joints may be summed up in the word "movement." It must always be an important question, therefore, in dealing with traumatic lesions of the articulations, how soon a joint will regain its normal mobility. How long must the part be kept at rest? How soon may we begin to move the limb?

In dealing with a sprain some will advise the patient "to walk it off"; others will enjoin prolonged rest. Now, as is so often the case, both methods are valuable in their place. We believe in rest, with accurate elastic pressure, with plenty of cotton wool, for two days, *i.e.*, until acute symptoms have sufficiently subsided to allow of the exercise of gentle massage and passive movement, gradually increased from day to day.

But it is in the management of fractures into and near joints that the greatest diversity of opinion exists on the subject of passive motion. In any doubtful injury to a joint it is always well to remember the possibility of fracture, and to treat the case as such, at any rate until the swelling and inflammation have somewhat abated. Here we find the great value of an anæsthetic. Fractures near a joint must often, from their very nature, be far more serious than fractures of the shaft of a long bone, and in discussing this question we may take the elbow as the best example of an osseously strong joint.

In 1868 Bigelow, in a clinical lecture, made a strong protest against the use of passive motion, which, as generally practised, sets up an active inflammation, and may be a serious injury to the part which is under repair. If, says Bigelow, when the splint is removed after the proper interval for repair, the arm can be flexed or extended through even a small arc, not with that deceptive springiness due to the elasticity of the ligaments, but in a way to satisfy the surgeon that the cartilages are sliding one upon the other, however little, the rest may be left to nature. But if the cartilages do not slide through even a small arc, owing to bony deformity, passive motion will not accelerate recovery. When there is bony deformity or projecting callus, passive motion does harm, and when the bones are in place and under supervision it is unnecessary.

Pilcher agrees with the position taken up by Verneuil, Allis, and Ray, in their recommendations to treat fractures of the elbow by prolonged application of an immovable apparatus. The elbow is thought by many to be specially prone to stiffness and anchylosis, and so much are these dreaded that early, and if necessary, persistent passive motion are considered by them as absolutely necessary. They claim that stiffness may be avoided by early passive motion, yet Allis's results, which will compare favourably with any, have been obtained without any passive motion earlier than the fourth week.

Stimson, in discussing this question, remarks that immobilization is not in itself a cause of anchylosis. It is, on the contrary, the most efficient agent we possess against the inflammation which is the primary cause of most stiffness, and passive motion is powerless to prevent anchylosis when conditions contributory thereto are present.

On the other hand, Lane says the pain of passive motion is greater in proportion as the movement is deferred to a late period. Hamilton has shown us how motion alone will not prevent union, but the movement of one fragment to and from its fellow is a frequent cause of non-union.

What conclusions, therefore, can we come to on this important question? Whilst the subject must be still considered *sub judice*, we believe that it must greatly depend upon the nature of the injury, and upon the character of the joint that is implicated. On the whole, we believe that it is a matter of accurate coäptation of the fragments.

